# Cell-death-inducing DFFA-like Effector B Contributes to the Assembly of Hepatitis C Virus (HCV) Particles and Interacts with HCV NS5A

**DOI:** 10.1038/srep27778

**Published:** 2016-06-10

**Authors:** Hua Cai, Wenxia Yao, Leike Li, Xinlei Li, Longbo Hu, Runming Mai, Tao Peng

**Affiliations:** 1 State Key Laboratory of Respiratory Disease, Guangzhou Hoffmann Institute, College of Basic Sciences, Guangzhou Medical University, Guangzhou, China; 2 Guangzhou Institutes of Biomedicine and Health, Chinese Academy of Sciences, Guangzhou, China; 3 The Brown Foundation Institute of Molecular Medicine at the University of Texas Health Science Center at Houston, Houston, TX, USA; 4 Division of Birth Cohort Study, Guangzhou Women and Children’s Medical Center, Guangzhou Medical University, Guangzhou, China

## Abstract

Hepatitis C virus (HCV) uses components of the very-low-density lipoprotein (VLDL) pathway for assembly/release. We previously reported that hepatocyte nuclear factor 4α (HNF4α) participates in HCV assembly/release through downstream factors those participate in VLDL assembly/secretion. Cell-death-inducing DFFA-like effector B (CIDEB) is an important regulator of the VLDL pathway. CIDEB is required for entry of HCV particles from cell culture (HCVcc), but the effects of CIDEB on the post-entry steps of the HCV lifecycle are unclear. In the present study, we determined that CIDEB is required for HCV assembly in addition to HCVcc entry. Furthermore, CIDEB interacts with the HCV NS5A protein, and the N terminus of CIDEB and the domain I of NS5A are involved in this interaction. Moreover, CIDEB silencing impairs the association of apolipoprotein E (ApoE) with HCV particles. Interestingly, CIDEB is also required for the post-entry stages of the dengue virus (DENV) life cycle. Collectively, these results indicate that CIDEB is a new host factor that is involved in HCV assembly, presumably by interacting with viral protein, providing new insight into the exploitation of the VLDL regulator CIDEB by HCV.

As a positive-strand RNA virus belonging to *Hepacivirus* of *Flaviviridae*, hepatitis C virus (HCV) hijacks host lipid metabolism during its life cycle^1^, including viral particle assembly/release. HCV assembly begins on the ER–lipid droplet (LD) surface, where the viral core protein, surface glycoproteins (E1 and E2), and viral RNA are assembled and packaged in a temporally and spatially regulated manner^2^,^3^,^4^. Nascent HCV particles then form by budding into the ER and fuse with pre-very-low-density lipoprotein (VLDL) particles to form lipo-viro particles (LVPs)^2^,^5^, or nascent HCV particles then form by budding of viral capsids into the ER lumen, incorporate cholesterol and triglycerides (TGs), and further bind ApoB and exchangeable apolipoproteins in a manner similar to lipoproteins to form LVPs^6^. The LVPs finally bud from the ER and transit via the secretory pathway^2^,^5^.

It has been widely discussed that HCV hijacks the VLDL secretory pathway to facilitate its assembly and secretion^7^,^8^,^9^. LDs, which are sources of VLDL lipidation, play a crucial role in HCV assembly^10^. Several VLDL key components, such as apolipoprotein E (ApoE), ApoC-I, ApoC-III, and ApoA-I, are involved in HCV assembly/release^8^,^11^,^12^,^13^,^14^,^15^,^16^,^17^. We previously reported that PLA_2_GXIIB, another VLDL component, also participates in HCV release^18^. One of the mechanisms by which HCV exploits the VLDL components is via protein-protein interactions between HCV proteins and VLDL components, as illustrated by the participation of ApoE in HCV assembly^12^,^19^,^20^. ApoE is an exchangeable apolipoprotein in VLDL assembly and participates in the formation of infectious HCV particles by interacting with HCV NS5A and E2 proteins^12^,^19^,^20^.

Cell-death-inducing DFFA-like effector B (CIDEB), a member of the CIDE protein family, is primarily expressed in liver tissues as well as in the small intestine^21^,^22^. As a CIDE family member, overexpression of CIDEB protein induces cell death, but the physiological function of CIDEB is more closely related to various lipid metabolic pathways, particularly the VLDL pathway^22^,^23^,^24^,^25^. For instance, CIDEB mediates VLDL lipidation and maturation by interacting with ApoB^24^; CIDEB is also required for the biogenesis of VLDL transport vesicles^25^ and for chylomicron lipidation in the small intestine^22^. CIDEB is transcriptionally regulated by hepatocyte nuclear factor 4α (HNF4α), the most abundant transcription factor in the liver; HNF4α is crucial for VLDL-mediated lipid transport and participates in HCV assembly/release^18^. As a key transcriptional coactivator of HNF4α, peroxisome proliferator-activated receptor-gamma coactivator-1α (PGC-1α) also regulates HCV production^26^, and PGC-1α stimulates VLDL assembly in a CIDEB-dependent manner^27^.

CIDEB is required for HCVcc entry into hepatocytes^28^; however, the effects of CIDEB on the post-entry steps of the HCV lifecycle are unclear. Based on the function and regulation of CIDEB, we hypothesized that CIDEB, as a VLDL regulator, is also involved in HCV assembly/release. In this study, we validated this hypothesis and determined that 1) CIDEB is a previously unrecognized requirement for HCV assembly; 2) CIDEB interacts with the HCV NS5A protein and regulates the association of HCV particles with ApoE; and 3) CIDEB also regulates the post-entry stages of the dengue virus (DENV) lifecycle.

## Results

### CIDEB contributes to the assembly of HCV particles

#### CIDEB is required for HCV production

**Figure 1:**
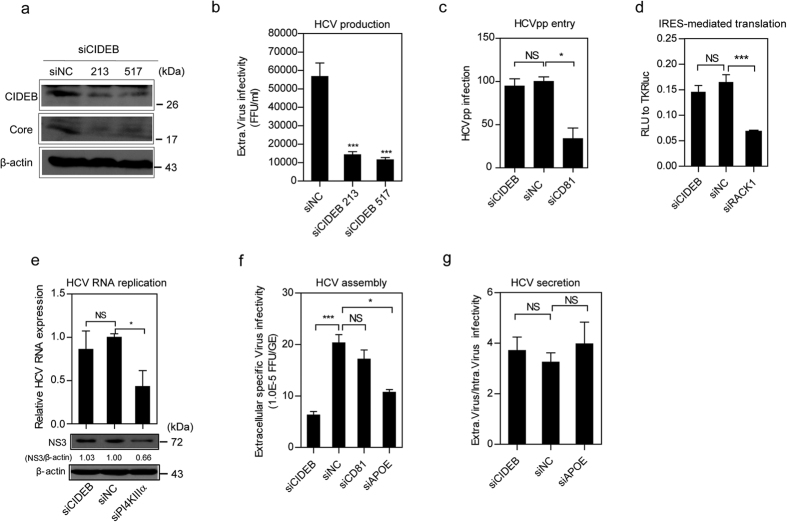
CIDEB is required for HCV production. (**a,b**) Huh7.5.1 cells were transfected with siCIDEB and then inoculated with HCV-Jc1EGFP (0.02 MOI) at 6 h post-transfection (hpt). Viral supernatants and cells were collected at 72 h post-infection (hpi). (**a**) Western blot analysis to detect CIDEB and HCV core protein expression. (**b**) Limiting dilution assay to determine the effect of CIDEB knockdown on infectious HCV titer. (**c**) The effect of the knockdown of the indicated genes on HCV entry was determined by an HCV pseudoparticle (HCVpp) assay. Huh7.5.1 cells were transfected with siRNA and then inoculated with HCVpp at 48 hpt. HCVpp infectivity was evaluated by FCM at 3 dpi. The values are presented relative to siNC after normalization by VSVGpp infectivity. (**d**) Effect of CIDEB knockdown on HCV IRES-mediated translation. Huh7.5.1 cells stably expressing T7 RNA polymerase and pretreated with siRNA for 48 h were transfected with the linearized SGR-GND-luciferase reporter plasmid, and luciferase expression was measured at 24 hpt. The pRL-TK-*Renilla* reporter plasmid served as a transfection control. (**e**) Effect of CIDEB knockdown on HCV NS3 protein and HCV RNA levels in Huh7.5.1-sgJFH2a cells. The ratio of the relative band intensity of NS3 normalized to β-actin was calculated. (**f,g**) Huh7.5.1 cells were transfected with siCIDEB and then inoculated with HCV-Jc1EGFP (0.02 MOI) at 6 hpt. Viral supernatants and cells were collected at 72 hpi. (**f**) Effect of CIDEB knockdown on the specific infectivity (the ratio of the extracellular HCV titer to extracellular HCV RNA) of HCV particles. FFU, focus-forming units; GE, genome equivalent. (**g**) Effect of CIDEB knockdown on the ratio of the extracellular infectious titer to the intracellular infectious titer. (**b–g**) The results are presented as the mean ± SD (*n* = 3 independent experiments; *P < 0.05, ***P < 0.001).

To determine the importance of CIDEB for HCV infection, we investigated whether decreasing CIDEB expression affects HCV production. Huh7.5.1 cells were transfected with CIDEB siRNAs and then inoculated with HCV-Jc1EGFP. Compared with siNC, siCIDEBs reduced HCV viral protein expression [as indicated by the HCV core protein (Fig. 1a) and the number of intracellular NS5A-positive cells (Fig. S1a)] and extracellular and intracellular HCV RNA levels (Fig. S1a). In addition, extracellular HCV infectivity also decreased, as observed in both the limiting dilution (Fig. 1b) and FCM (Fig. S1a) assays. No siRNA-related toxicity effect was observed (data not shown).

CD81^29^, RACK1^29^, PI4KIIIα^31^,^32^,^33^, and ApoE^8^,^12^,^17^,^34^ are important cellular factors in HCV entry, IRES-mediated translation, RNA replication, and assembly, respectively. To determine the specific stage of the HCV life cycle that requires CIDEB, a panel of siRNAs targeting these genes was used. Consistent with a previous report, CIDEB silencing did not affect HCV pseudoparticle (HCVpp) infection (Fig. 1c) but did affect HCVcc entry (Fig. S1c). To determine whether CIDEB is required for HCV IRES-mediated translation, Huh7.5.1 cells stably expressing T7 RNA polymerase were treated with siRNAs, followed by transfection with the linearized HCV subgenomic replicon (SGR-GND-Luc) downstream of the T7 RNA promoter. SGR-GND is defective in HCV RNA replication but is competent in HCV protein translation^35^. As shown in Fig. 1d, siCIDEB treatment did not reduce the level of luciferase expression. As a positive control, silencing of RACK1 (which is important for HCV IRES-mediated translation^30^) significantly impaired luciferase expression. This result suggests that CIDEB does not affect HCV IRES-dependent translation.

To determine whether CIDEB is required for HCV RNA replication, siCIDEB was transfected into Huh7.5.1 cells harboring HCV subgenomic replicons: Huh7.5.1-sgJFH2a for genotype 2a and Huh7-sgHCV1b for genotype 1b. CIDEB knockdown had no effect on intracellular HCV RNA and NS3 protein levels in both Huh7.5.1-sgJFH2a cells (Fig. 1e) and Huh7-sgHCV1b cells (data not shown). By contrast, as a positive control, siPI4KIIIα significantly decreased HCV RNA and NS3 protein expression levels (Fig. 1e). This result suggests that CIDEB does not affect HCV RNA replication.

To further investigate whether CIDEB is involved in the late stage of the HCV life cycle, we calculated the specific infectivity (infectious titer divided by the HCV RNA copy number) and the ratio of the extracellular HCV titer to the intracellular HCV titer, as previously described^18^,^26^. The specific infectivity was calculated to evaluate the efficiency of HCV assembly^36^, whereas the ratio of the extracellular titer to the intracellular titer was used to evaluate the level of viral particle secretion^18^,^26^. Results showed that similar to siApoE, siCiDeB significantly reduced extracellular HCV specific infectivity whereas siCD81 had no effect (Fig. 1f), and that both siCiDeB and siApoE had no effect on the ratio of the extracellular titer to the intracellular titer (Fig. 1g). These results suggest that CIDEB regulates HCV particle assembly but not secretion.

Collectively, these results indicate that, in addition to entry^28^, CIDEB also affects HCV assembly.

#### CIDEB is involved in the assembly step of the HCV life cycle

**Figure 2:**
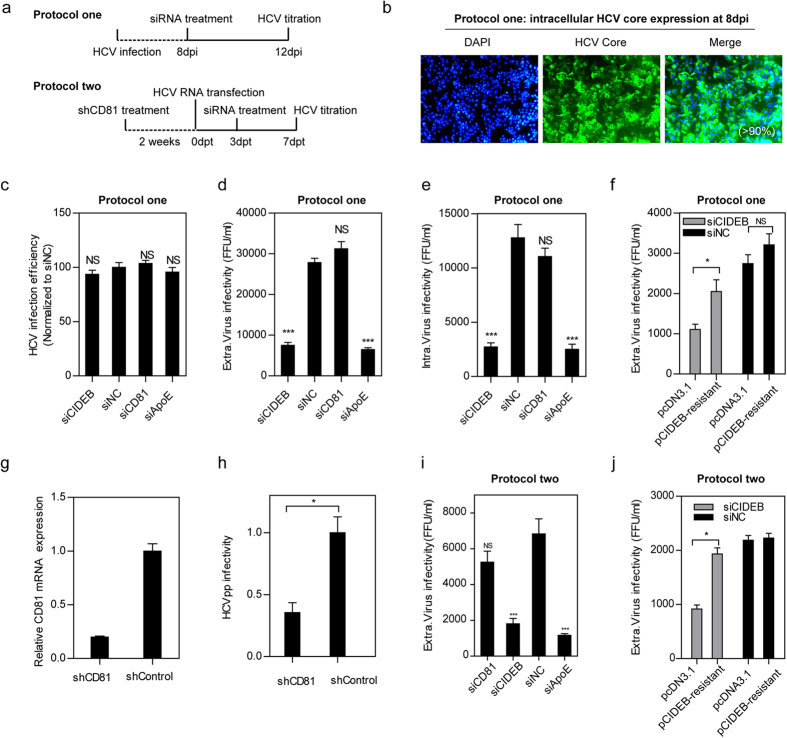
CIDEB is required for the assembly of HCV particles. (**a**) Schematic of the two protocols used to determine the effect of CIDEB silencing on infectious HCV particle assembly. In protocol one, Huh7.5.1 cells were infected with HCV-Jc1EGFP (0.02 MOI) for 8 days, re-seeded, and transfected with siRNA. Viral supernatants and cells were collected at 12 dpi. In protocol two, Huh7.5.1 cells were transduced with shRNA targeting CD81, and pools of cells with stable shCD81 expression (named shCD81-transduced Huh7.5.1 cells) were established by puromycin selection. shCD81-transduced Huh7.5.1 cells were then transfected with HCV-Jc1 RNA for 3 days, re-seeded, and transfected with siRNA. Viral supernatants and cells were collected at 7 dpt. (**b–f**) The effect of CIDEB silencing on HCV assembly was evaluated by protocol one. (**b**) The intracellular core expression in HCV-infected Huh7.5.1 cells was detected by IFA at 8 dpi. (**c**) FCM analysis of the percentage of intracellular NS5A-EGFP-positive cells. (**d**) Analysis of extracellular HCV infectivity (FFU/ml). (**e**) Analysis of intracellular HCV infectivity (FFU/ml). (**f**) Persistently HCV-infected Huh7.5.1 cells were co-transfected with siCIDEB and plasmid expressing siRNA-resistant CIDEB at 8 dpi, and the HCV titer in the supernatants was then evaluated at 12 dpi. (**c–f**) The results are presented as the mean ± SD (*n* = 3 independent experiments; *P < 0.05, ***P < 0.001). (**g–j**) The effect of CIDEB silencing on HCV assembly was evaluated by protocol two. (**g**) qRT-PCR was performed to determine the CD81 mRNA level (normalized to shControl after normalization to 18S rRNA). (**h**) HCVpp infectivity in shCD81 and shControl cells was evaluated by FCM at 3 dpi. (**i**) The extracellular HCV infectivity was determined. (**j**) shCD81-transduced Huh7.5.1 cells were co-transfected with siCIDEB and a plasmid expressing siRNA-resistant CIDEB at 3 dpt, and the HCV titer in the supernatants was evaluated at 7 dpt. (**h–j**) The results are presented as the mean ± SD (*n* = 3 independent experiments; *P < 0.05, ***P < 0.001).

To further confirm the importance of CIDEB for HCV assembly, we employed two additional cell lines: persistently HCV-infected Huh7.5.1 cells and shCD81-transduced Huh7.5.1 cells (Fig. 2a). We defined Huh7.5.1 cells from 8 days post-infection (dpi) with HCV as persistently HCV-infected cells; these cells exhibited a relatively constant infection efficiency of >90%, as evidenced by the percentage of core-positive cells (Fig. 2b). The persistently infected cells and shCD81-transduced cells did not permit HCV re-infection; therefore, these cells were used to analyze the importance of CIDEB for HCV assembly without interference from HCVcc entry.

HCV-Jc1EGFP persistently infected cells were transfected with CIDEB siRNA. Cell lysates and culture supernatants were collected and analyzed at 4 dpt (Fig. 2a). The percentage of NS5A-EGFP-positive cells did not change upon siCIDEB, siCD81, or siApoE treatment, suggesting that HCV reinfection did not happen in HCV-Jc1EGFP persistently infected cells (Fig. 2c). In addition, similar to siApoE, siCIDEB markedly decreased extracellular and intracellular HCV infectivity, whereas siCD81 had no effect (Fig. 2d,e) (as shown in Fig. S1d, siCD81 pre-treatment decreased HCV production when naïve Huh7.5.1 cells were infected with HCV-Jc1EGFP, suggesting that siCD81 functioned properly). Moreover, exogenous overexpression of CIDEB encoded by a siCIDEB-resistant gene rescued extracellular HCV infectivity impaired by CIDEB knockdown (Fig. 2f). Furthermore, expression of a CIDEB TALEN (transcription activator-like effector nucleases) pair significantly decreased extracellular HCV infectivity in HCV-Jc1EGFP persistently infected cells (Fig. S1e–h), confirming that CIDEB is responsible for the decreased HCV infectivity.

To confirm the above observation, an alternative approach was taken. Two weeks after shCD81-transduction, Huh7.5.1 cells were transfected with full-length HCV RNA and then treated with siRNA at 3 dpt. Cells and supernatants were collected and analyzed at 7 dpt (Fig. 2a). shCD81 treatment efficiently decreased CD81 mRNA levels and inhibited HCVpp infectivity (Fig. 2g,h), suggesting that shCD81 functioned properly. Both siCIDEB and siApoE decreased extracellular HCV infectivity (as shown in Fig. 2i; further challenge in shCD81-transduced Huh7.5.1 cells with siCD81 had no effect, suggesting that shCD81 transduction did not permit HCV re-infection). Exogenous overexpression of resistant CIDEB rescued the decrease in extracellular HCV infectivity induced by CIDEB knockdown (Fig. 2j).

These results confirm that CIDEB is involved in HCV assembly.

### CIDEB interacts with NS5A

Because CIDEB is located on LDs and the smooth ER^24^, where HCV proteins important for HCV assembly, namely core and NS5A, are anchored^2^,^5^, we further investigated whether CIDEB functions through an interaction with HCV proteins.

#### CIDEB co-localizes with NS5A

**Figure 3:**
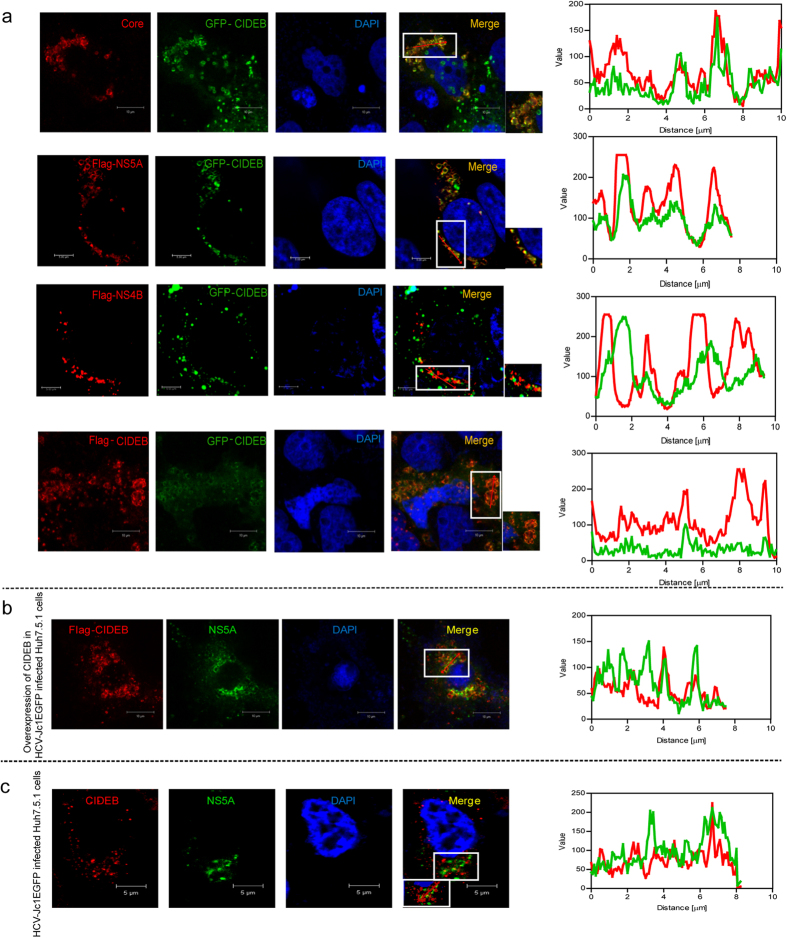
Analysis of the co-localization of CIDEB with HCV NS5A. (**a**) The co-localization of GFP-CIDEB with HCV proteins (Core, Flag-NS5A, and Flag-NS4B) was analyzed by confocal microscopy. The magnification of the framed section of the merged images is presented at the right of the merged images. (**b**) Co-localization of exogenous Flag-CIDEB with endogenous NS5A-EGFP in HCV-Jc1EGFP-infected Huh7.5.1 cells. (**c**) Co-localization of endogenous CIDEB with endogenous NS5A-EGFP in HCV-Jc1EGFP-infected Huh7.5.1 cells. The insets at the lower left of the merged images show the boxed areas with an arrow. (**a–c**) Arrows indicate the selected lines in the RGB line profiles to the right of each row.

To determine whether CIDEB co-localizes with HCV proteins, GFP-CIDEB was co-expressed with each individual HCV protein and examined by confocal microscopy. Because CIDEB exists as a homodimer^37^, the co-localization of Flag-CIDEB and GFP-CIDEB was used as a positive control (Fig. 3a). As shown in Fig. 3a, CIDEB co-localized with HCV NS5A and the core protein but not with the NS4B protein. The co-localization of NS5A with CIDEB was further confirmed by subjecting HCV-Jc1EGFP-infected cells to a co-localization assay. Both exogenous Flag-CIDEB (Fig. 3b) and endogenous CIDEB (Fig. 3c) co-localized with endogenous NS5A-EGFP. We concluded from these results that CIDEB co-localizes with HCV NS5A.

#### CIDEB interacts with NS5A

**Figure 4:**
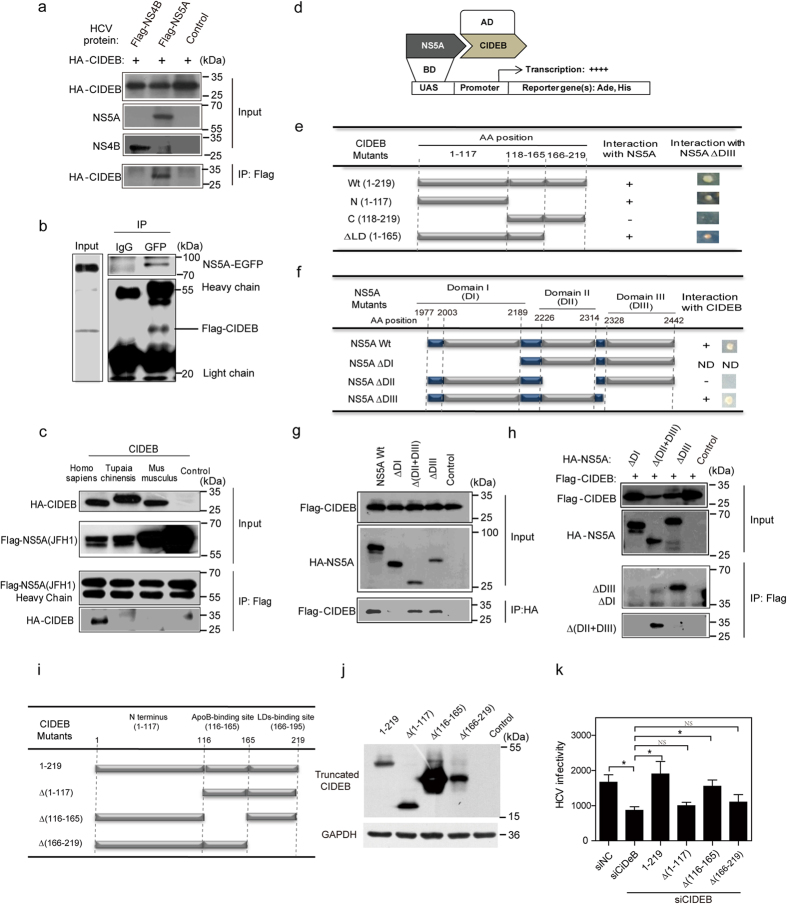
CIDEB interacts with the HCV NS5A protein, and the N terminus of CIDEB and domain I of NS5A are essential for the CIDEB-NS5A interaction. (**a**) Co-IP assay to determine the interaction of exogenous HA-CIDEB with Flag-NS5A and Flag-NS4B in HEK293T cells. Cell lysates were subjected to IP with an anti-Flag antibody and then immunoblotted with an anti-HA antibody. (**b**) Co-IP assay to determine the interaction of exogenous Flag-CIDEB with endogenous NS5A-EGFP in HCV-Jc1EGFP-infected Huh7.5.1 cells transfected with pReceiver-Flag-CIDEB. (**c**) Co-IP assay to determine the interaction of exogenous CIDEB from different species (*Homo sapiens*, *Tupaia chinensis*, and *Mus musculus*) with Flag-NS5A (from HCV-JFH1) in HEK293T cells. (**d**) The principle of the yeast two-hybrid (Y2H) assay: AH109 transfected with HCV NS5A mutants mated with Y187 transfected with CIDEB mutants; interaction between two proteins is indicated by the activation of the reporter genes HIS3 and ADE, which allows the mated yeast cells to grow on plates containing SD/–Ade/–His/–Leu/–Trp (+ + + +). UAS: upstream activating sequence; AD: activation domain; BD: DNA-binding domain. (**e**) Schematic of the tested CIDEB constructs and screening results for the CIDEB-NS5A interaction. (**f**) Schematic of the tested NS5A constructs and screening results for the CIDEB-NS5A interaction. ND: cannot be determined due to autoactivation. (**g,h**) Co-IP assays to identify the specific domain of NS5A responsible for the CIDEB-NS5A interaction. HEK293T cells were co-transfected with the expression vectors for Flag-CIDEB and truncated HA-NS5A. (**g**) The cell lysates were subjected to IP with an anti-HA antibody and then immunoblotted with an anti-Flag antibody. (**h**) The cell lysates were subjected to IP with an anti-Flag antibody and then immunoblotted with an anti-HA antibody. (**i–k**) The effect of siRNA-resistant truncated CIDEB on HCV production in siCIDEB-pretreated Huh7.5.1 cells. (**i**) Schematic of CIDEB and its truncations. (**j**) Western blot analysis to detect the overexpression of siRNA-resistant CIDEB and its truncations. (**k**) The effect of siRNA-resistant truncated CIDEB overexpression on the extracellular HCV titer in siCIDEB-pretreated Huh7.5.1 cells. The results are presented as the mean ± SD (*n* = 3 independent experiments; *P < 0.05).

To determine whether CIDEB directly interacts with NS5A, co-immunoprecipitation experiments were performed. HEK293T cells were co-transfected with HCV proteins and CIDEB. The cell lysates were subjected to IP using an anti-Flag or anti-HA antibody or anti-GFP antibody and then immunoblotted using anti-HA or anti-Flag or anti-NS3 antibodies. CIDEB co-immunoprecipitated with NS5A (Fig. 4a and Fig.S2a) and NS2 (Fig.S2a) but not with Core (Fig. S2a), NS3 (Fig. S2a), NS4B (Fig. 4a), or NS5B (Fig. S2a), indicating that CIDEB interacts with NS5A in addition to the previous reported NS2^37^. IP assays using HCV-Jc1EGFP-infected cells transfected with Flag-CIDEB further confirmed that CIDEB also interacts with NS5A in HCV infected cells (Fig. 4b).

To determine whether the NS5A-CIDEB interaction is conserved among different genotypic NS5A or among different species of CIDEB, HEK293T cells were co-transfected with the expression vectors pReceiver-Flag-NS5A (from genotype 2a or 1b) and pReceiver-HA-CIDEB (from human, primate tree shrew or rodent mouse). The cell lysates were subjected to IP using an anti-Flag antibody and then immunoblotted using an anti-HA antibody. As shown in Fig. 4c and S2b, human HA-CIDEB co-immunoprecipitated with Flag-NS5A from both genotype 2a (JFH1) and genotype 1b (Con1), whereas tree shrew HA-CIDEB and mouse HA-CIDEB did not co-immunoprecipitate with Flag-NS5A from either genotype. These results indicate that human CIDEB interacts with HCV NS5A, whereas tree shrew CIDEB and mouse CIDEB do not interact with HCV NS5A.

Collectively, these results indicate that CIDEB interacts with HCV NS5A.

#### The N terminus of CIDEB and domain I of NS5A are involved in the CIDEB-NS5A interaction

To further explore the domains responsible for the CIDEB-NS5A interaction, a yeast two-hybrid (Y2H) assay was performed (Fig. 4d). As indicated in Fig. 4e, analysis of various CIDEB mutants revealed that the N terminus of CIDEB is required for the interaction with NS5A. NS5A deletion mutants were also evaluated to identify the NS5A domains essential for the NS5A-CIDEB interaction. Deletion of domain II eliminated the NS5A-CIDEB interaction, whereas deletion of domain III did not affect the NS5A-CIDEB interaction (Fig. 4f and S2c). NS5A-∆DI was autoactivated in this Y2H system; thus, we could not evaluate its interaction with CIDEB (Fig. 4f and S2c). We further performed co-IP assays to identify which domain of NS5A accounts for the CIDEB-NS5A interaction. As shown in Fig. 4g,h, both NS5A-∆(DII + DIII) and NS5A-∆DIII co-immunoprecipitated with Flag-CIDEB, whereas NS5A-∆DI did not co-immunoprecipitate with Flag-CIDEB, suggesting that the DI domain is required for the CIDEB-NS5A interaction. Taken together, the Y2H results and the co-IP results indicate that the N terminus of CIDEB and domain I of NS5A are involved in the CIDEB-NS5A interaction (summarized in Fig. S2c).

The Y2H analysis also suggested that the NS5A-CIDEB interaction is not restricted to genotype 2a because the interaction was also observed with NS5A from genotype 1b (HCV-Con1) (Fig. S2c). The HCV core and NS3 did not interact with CIDEB in the Y2H system (data not shown).

These results collectively indicate that CIDEB interacts with NS5A and that the N terminus of CIDEB and domain I of NS5A are involved in the CIDEB-NS5A interaction (summarized in Fig. S2c).

#### The NS5A-interaction domain and LD-binding domain of CIDEB are required for HCV production

To identify the specific domains of CIDEB that are functionally important for HCV production, Huh7.5.1 cells were transfected with siCIDEB to knockdown the endogenous CIDEB protein. These cells were then transfected with plasmids encoding truncated forms of CIDEB that are siCIDEB-resistant. Exogenous overexpression of siRNA-resistant full-length CIDEB rescued extracellular HCV infectivity impaired by CIDEB knockdown, whereas expression of CIDEB ∆(1–117) did not rescue HCV production, suggesting that the N-terminal region that interacts with NS5A is required for HCV production (Fig. 4i–k). In addition, deletion of the LD-binding domain (aa 166–219) within CIDEB abolished its ability to rescue HCV production, whereas deletion of the ApoB-interaction domain (aa 116–165) rescued HCV production, suggesting that the LD-binding domain but not the ApoB-interaction domain of CIDEB is required for HCV production (Fig. 4k). Collectively, these results suggest that the NS5A-interaction domain and LD-binding domain of CIDEB are required for HCV production.

### CIDEB silencing impairs the association of HCV particles with ApoE and disrupts the NS5A-ApoE interaction

#### CIDEB silencing impairs the association of HCV particles with ApoE

**Figure 5:**
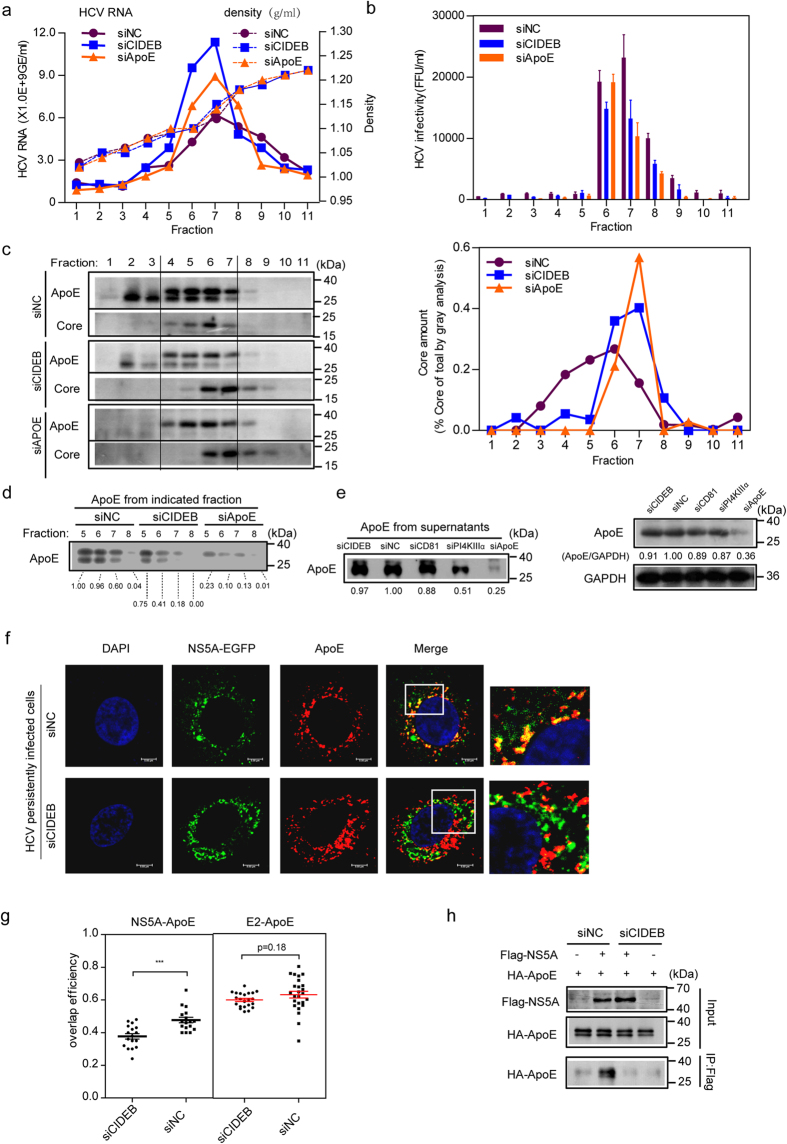
CIDEB silencing impairs the association of HCV particles with ApoE and disrupts the NS5A-ApoE interaction. (**a–c**) Concentrated culture supernatants from HCV persistently infected Huh7.5.1 cells treated with siRNAs were subjected to 20–60% sucrose gradients and equilibrium ultracentrifugation. Eleven fractions were harvested from the top of the sucrose gradients and used to determine the density, HCV RNA levels, infectivity titer, and HCV core amounts. (**a**) The buoyant density of sucrose was plotted with the HCV RNA level as measured by qRT-PCR in each fraction. (**b**) The HCV infectivity of each fraction was evaluated. (**c**) Left: Western blot analysis of the ApoE and core protein levels in each fraction of the sucrose gradient. The right panel displays the relative core-to-total-core amounts as detected in all fractions. (**d**) Western blot analysis of ApoE from the indicated fractions of the sucrose gradient those were rich in HCV infectious particles. (**e**) Western blot analysis of total supernatants ApoE (left) and cell lysates ApoE (right). (**f**) The effect of CIDEB silencing on the co-localization of NS5A-EGFP with endogenous ApoE in HCV- Jc1EGFP persistently infected Huh7.5.1 cells. The magnification of the frame section of merged images is presented at the right of the merged images. (**g**) The effect of CIDEB silencing on NS5A-ApoE and E2-ApoE co-localization was evaluated by overlap efficiency. (**h**) The effect of CIDEB silencing on the NS5A-ApoE interaction. Huh7.5.1 cells were transduced with Flag-NS5A and HA-ApoE lentivirus for 48 h and then treated with siNC or siCIDEB for 72 h. Cell lysates were subjected to IP with an anti-Flag antibody and then immunoblotted with an anti-HA antibody.

We next characterized the extracellular HCV particles produced from siCIDEB-treated HCV-Jc1EGFP persistently infected cells by sucrose density gradient centrifugation. Because ApoE is a key component of infectious HCV particles and affects HCV assembly, siApoE-treated cells were used as a positive control. In most of the fractions, higher HCV RNA copy numbers (Fig. 5a) and lower HCV infectivity (Fig. 5b) were observed in siCIDEB and siApoE cells compared with siNC cells. This result is consistent with a previous report in which ApoE silencing enhanced the extracellular HCV RNA content^12^. Although the density distribution of the HCV infectious particles produced from siCIDEB cells was similar to that of particles produced from siNC-treated cells, we observed a shift toward a higher density of viral core protein released from both siCIDEB and siApoE cells (Fig. 5c). These results indicate that CIDEB silencing produces higher levels of core protein and HCV RNA, which is generally not infectious in HCV persistently infected cells.

Reduced ApoE expression from fractions 4 to 7 released from siCIDEB and siApoE cells was observed compared with siNC cells (Fig. 5c). This reduced expression was most apparent when ApoE expression from fractions 6 to 8 was visualized in the same gel (Fig. 5d). However, ApoE expression in the total supernatant and cell lysates were similar for siCIDEB-treated and siNC cells (Fig. 5e). These results suggest that CIDEB silencing affects the amount of ApoE associated with HCV particles without affecting the total amounts of ApoE secreted in the supernatant.

Collectively, these results indicate that CIDEB silencing impairs the association of HCV particles with ApoE.

#### CIDEB silencing disrupts the NS5A-ApoE interaction

Previous studies have suggested that HCV NS5A and E2 interact with ApoE and are involved in ApoE recruitment^12^,^19^,^38^. To determine whether CIDEB is important for these interactions, we analyzed the effect of siCIDEB treatment on co-localization of NS5A and E2 with ApoE. siCIDEB impaired the co-localization of NS5A-EGFP with endogenous ApoE in HCV-Jc1EGFP persistently infected Huh7.5.1 cells (Fig. 5f,g) but had no effect on ApoE-E2 co-localization (Fig. S3). Moreover, Huh7.5.1 cells transduced with Flag-NS5A and HA-ApoE lentivirus were treated with siNC or siCIDEB, and CIDEB silencing impaired exogenous NS5A-ApoE interaction in the co-IP analysis (Fig. 5h). These results suggest that CIDEB is involved in the NS5A-ApoE interaction.

### CIDEB is required for the post-entry stages of the DENV lifecycle

**Figure 6:**
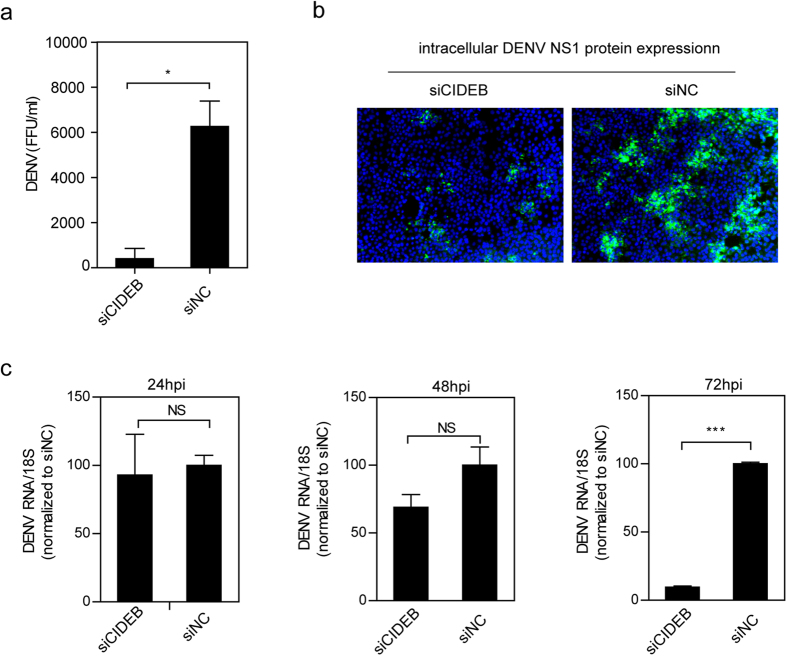
CIDEB is required for DENV production. Huh7.5.1 cells were transfected with siCIDEB and then inoculated with DENV at 48 hpt. (**a**) Limiting dilution assay to determine the effect of CIDEB knockdown on the DENV titer at 72 hpi. (**b**) The effect of CIDEB silencing on DENV NS1 expression at 72 hpi was quantified by IFA assay. (**c**) The intracellular DENV RNA level at the indicated times was determined by qRT-PCR. (**a**,**c**) The results are presented as the mean ± SD (*n* = 3 independent experiments; *P < 0.05, ***P < 0.001).

In addition to HCV, other viruses of the genus *Flavivirus*, such as DENV, interact with host lipid metabolism^39^. To determine whether CIDEB utilization is unique to HCV or also occurs in DENV, DENV production was analyzed in Huh7.5.1 cells treated with siRNA. CIDEB silencing decreased extracellular DENV infectivity (Fig. 6a). NS1 protein staining was weaker in CIDEB-knocked down cells, indicating lower DENV replication (Fig. 6b). To determine which steps of the DENV lifecycle are affected by CIDEB, time-course analysis was performed to monitor the DENV infection efficiency. Compared with siNC cells, DENV RNA levels were unchanged in siCIDEB treated cells at 24 and 48 hpi but markedly lower at 72 hpi (Fig. 6c). These results indicate that CIDEB is required for the post-entry stages of the DENV life cycle.

## Discussion

This study is the first to report that CIDEB, a VLDL regulator, contributes to HCV assembly and interacts with the HCV NS5A protein.

We demonstrated that CIDEB is involved in the assembly step of the HCV lifecycle. This study provides the following lines of evidence to demonstrate that CIDEB is involved in HCV assembly: 1) decreased extracellular HCV-Jc1 specific infectivity upon siCIDEB treatment (Fig. 1f); 2) decreased intracellular and extracellular HCV infectivity in HCV-Jc1EGFP persistently infected Huh7.5.1 cells upon CIDEB silencing (Figs 2d,e and S1h); 3) decreased HCV infectivity upon CIDEB silencing in shCD81-transduced Huh7.5.1 cells (Fig. 2i); 4) Exogenous overexpression of siCIDEB-resistant CIDEB rescued extracellular HCV infectivity impaired by CIDEB knockdown in HCV-Jc1EGFP persistently infected cells (Fig. 2f) and shCD81-transduced Huh7.5.1 cells (Fig. 2j); 5) alteration of the biophysical characteristics of HCV particles by siCIDEB (Fig. 5).

Three laboratories have recently focused on the effect of CIDEB on the HCV life cycle. Wu *et al*. demonstrated that CIDEB is required for HCVcc entry into hepatocytes and does not affect HCV RNA replication in the HCV subgenomic system^28^. Singaravelu *et al*. demonstrated that siCIDEB treatment does not affect HCV RNA replication in Huh7 cells harboring an HCV subgenomic replicon. This study is the first to report that CIDEB contributes to HCV assembly and that CIDEB interacts with the HCV NS5A protein. In summary, CIDEB affects HCV entry and assembly but not HCV RNA replication in Huh7 and derived cells. The demonstrated contribution of siCIDEB treatment on HCV RNA replication in human-serum-differentiated hepatoma cells observed by Singaravelu *et al*.^40^ may reflect differences between serum-differentiated hepatoma cells and Huh7 cells. In addition, Lee *et al*. and Wu *et al*. from the same group found that HCV infection down-regulates CIDEB protein and also investigated the mechanism and biological consequence of HCV-induced downregulation of CIDEB^41^. They found that HCV down-regulates CIDEB by inducing CIDEB protein degradation, most likely through proteolytic cleavage, and further demonstrated that knockout of CIDEB in Huh7.5 cells reduces TGs secretion and VLDL lipidation and stabilizes cytoplasmic LDs in a manner similar to HCV infection, which may contributes to hepatic steatosis in the setting of HCV infection^41^. Considering the important role of CIDEB in LDs stabilization and VLDL secretion, it is highly pertinent that CIDEB plays a role in HCV lipoviral particle assembly.

**Figure 7:**
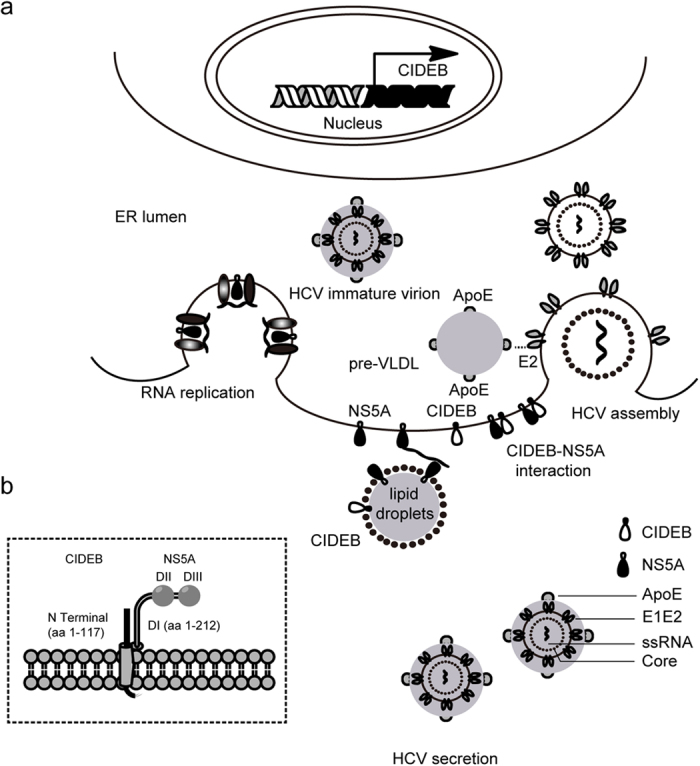
Summary of the association of HCV with CIDEB in the HCV lifecycle. (**a**) CIDEB participates in HCV assembly and interacts with NS5A. By localizing on the smooth ER and LDs by its C terminus, the N terminus of CIDEB may interact with NS5A to influence the NS5A-ApoE interaction, which further affect the association of HCV particles with ApoE. (**b**) A proposed model for the interaction between CIDEB and NS5A. The N terminus of CIDEB and domain I of NS5A are involved in the CIDEB-NS5A interaction.

CIDEB is required for VLDL assembly/release. In this study, CIDEB was identified as a novel host factor that facilitates HCV assembly and interacts with NS5A; CIDEB silencing impaired the NS5A-ApoE interaction and the association of ApoE with the HCV particle and resulted in the production of higher levels of core protein and HCV RNA, which are generally not infectious. Fusions of pre-VLDL with premature HCV particles promote the low density and high infectivity of HCV particles^5^. These results suggest that HCV may use CIDEB and/or the NS5A-CIDEB interaction to promote the fusion of pre-VLDL with premature HCV particles to form infectious HCV particles (Fig. 7). CIDEB is predominantly located on LDs and secondarily on the smooth ER by its C terminus^24^. Therefore, by localizing on the smooth ER and LDs by its C terminus, the N terminus of CIDEB may interact with NS5A to influence the NS5A-ApoE interaction and further affect the fusion of pre-VLDL with premature HCV particles (Fig. 7). However, the detailed mechanisms should be further investigated.

NS5A is a multifunctional protein, and its functions are presumably dependent on interactions with various host factors. In this study, CIDEB was identified as a novel NS5A-interacting protein, as determined by confocal microscopy analysis, co-immunoprecipitation, and Y2H analysis. Notably, CIDEB silencing impaired the NS5A-ApoE interaction (Fig. 5f–h). These results suggest that CIDEB does not compete with ApoE to interact with NS5A, which is consistent with that different domains of NS5A interact with CIDEB and ApoE: domain I of NS5A interacts with CIDEB, whereas domain III of NS5A interacts with ApoE^12^. In addition, the correlation between reduced association of ApoE with HCV particles (Fig. 5c,d) and decreased NS5A-ApoE interaction (Fig. 5f,h) suggested that CIDEB silencing impaired the association of HCV particles with ApoE probably by disrupting NS5A-ApoE interaction. Moreover, CIDEB co-localizes rather than interacts with ApoE (Fig. S3b,c). The NS5A-CIDEB interaction may bring NS5A closer to ApoE to promote the NS5A-ApoE interaction, further contributing to the assembly of HCV infectious particles.

The N terminus (aa 1–117) of CIDEB is required for the NS5A-CIDEB interaction. The structure of CIDEB (aa 1–117) has been analyzed by NMR spectroscopy^42^. Both ends of CIDEB (aa 1–117) exhibit conformational flexibility, and the internal portion of CIDEB (aa 1–117), which consists of two oppositely charged regions, may be involved in the CIDEB-NS5A interaction. In contrast to mouse and tree shrew CIDEB, human CIDEB interacts efficiently with the HCV NS5A protein (Figs 4c and S2b). We also observed a weak co-immunoprecipitation of tree shrew CIDEB with NS5A in Fig. 4C, which suggests a weak interaction. Differences in the CIDEB-coding sequences among *Homo sapiens*, *Tupaia belangeri chinensis*, and *Mus musculus* probably determine the NS5A-CIDEB interaction (Fig. S2b). The gradually decreased detection of the interaction of NS5A with CIDEB from *Homo sapiens*, *Tupaia belangeri chinensis*, and *Mus musculus* is consist with the gradually reduced susceptibility of these species to HCV infection, which suggested that CIDEB is an important determinant for the HCV host tropism. In addition to NS5A, NS2 has also been reported to interact with CIDEB^37^. A previous two-hybrid analysis^37^ demonstrated that the amino acids at positions 135 to 139 of NS2 are responsible for the CIDEB-NS2 interaction. We performed IP analysis and detected the interaction between NS2 and CIDEB in an overexpression system (Fig. S2a). Considering that NS2 plays an important role in HCV assembly^43^,^44^,^45^ as well as CIDEB, the NS2-CIDEB interaction might contribute to HCV assembly. However, further study is needed to confirm this hypothesis.

As members of the family *Flaviviridae*, both DENV and HCV interact with host lipid metabolism^10^,^39^. The present study demonstrated that CIDEB is also required for DENV production (Fig. 6). This result suggests that CIDEB may be involved in other RNA virus life cycles in which lipid metabolism plays a critical role. However, further investigation is required to confirm this hypothesis.

In summary, we identified a novel function of CIDEB in HCV assembly and interaction with NS5A. Our results demonstrate that CIDEB silencing impairs the ApoE-NS5A interaction, which may underlie the decrease of ApoE amounts in HCV particles and the invalid assembly of HCV particles. These results provide new insights into the exploitation of the VLDL regulator CIDEB by HCV.

## Methods

### Cell culture

HEK293T (ATCC), Huh7.5.1 (from Dr. Francis V. Chisari, The Scripps Research Institute, La Jolla, California, USA), Huh7-sgHCV1b and Huh7.5.1-sgJFH2a cells were maintained as previously reported^18^.

### Plasmids

The pRlenti vector was used to generate the following expression plasmids: HA-APOE, Flag-NS4B and Flag-NS5A. HA-CIDEB (*Homo sapiens*: NP_055245.2, *Mus musculus*: NP_034024.2, and *Tupaia belangeri chinensis*: XP_006144636.1), Flag-CIDEB and GFP-CIDEB were constructed by cloning CIDEB-encoding fragments into the following respective plasmids: pReceiver-M06 (3 × HA) pReceiver-M12 (3×Flag), and pReceiver-M29 (GFP). Flag-NS5A (from JFH1 and Con1) and Flag-Core were constructed with pReceiver-M12 plasmids. HA-NS5A, truncated HA-NS5A and HA-NS5B were constructed with pReceiver-M06 (3×HA). GFP-NS2 was constructed with pReceiver-M29 (GFP).

### Virus

The adapted HCV-Jc1EGFP (the EGFP gene was inserted into the C terminus of the NS5A-encoding sequence in the HCV-Jc1 genome) was prepared as previously described^18^. Dengue type 2 virus strain 43 was provided by Dr. Zhao Wei (Southern Medical University, Guangzhou, China). Lentiviral particles were produced as previously described^46^ by transfecting HEK293T cells with the pRlenti construct, the packaging psPAX2 construct (Addgene plasmid 12260), and a construct that expressed the glycoprotein of vesicular stomatitis virus pMD2.G (Addgene plasmid 12259).

### Antibodies and reagents

The following antibodies were used: anti-HCV core antibody (C7-50, Abcam), anti-HCV NS3 antibody (H23, Abcam), monoclonal mouse GFP antibody (AG281, Beyotime), monoclonal mouse anti-Flag antibody (F1804, Sigma), monoclonal mouse HA antibody (AH158, Beyotime), polyclonal rabbit HA Antibody (Sc-805, Santa Cruz), anti-DENV NS1 antibody (ab41616, Abcam), anti-β-actin antibody (BioVision), anti-CIDEB monoclonal antibody (M01, Abnova), anti-ApoE antibody (10817-RP02, Sino Biological), polyclonal anti-CIDEB antibody (LS-C119539, Lifespan), goat anti-mouse antibody (Chemicon), goat anti-rabbit antibody (Chemicon) conjugated with horseradish peroxidase, Cy3-labeled goat anti-mouse IgG (H + L) (Beyotime), Alexa Fluor 647-labeled goat anti-rabbit IgG (Beyotime), Alexa Fluor 555-labeled donkey anti-rabbit IgG (H + L) (Beyotime) and Alexa Fluor 488-labeled donkey anti-mouse IgG (H + L) (Invitrogen).

The siRNA sequences specifically targeting HCV, CD81, PI4KIIIα, ApoE and RACK1 were previously reported^18^,^47^. The target sequences of the CIDEB siRNA were as follows: 5′-AGAGGAGGATGGAACTGCA-3′ (siCIDEB 213) and 5′-AGTACTCAGGGAGCTCC-3′ (siCIDEB 517).

### Virus titration assay

HCV titers in cell lysates or viral supernatants were quantified as previously described^26^. In brief, two assays were performed to determine HCV titers. To titrate supernatant HCV-Jc1 titers, viral supernatants were clarified and inoculated in Huh7.5.1 cells by end-point dilution. Stained foci were counted and used to calculate the titer in focus-forming units (FFU)/ml. Flow cytometry (FCM) was also conducted for supernatant HCV-Jc1EGFP titration. Approximately 5 μl of viral supernatant was used to inoculate Huh7.5.1 cells in 24-well tissue culture plates. The infected cells were quantified by FCM at 72 h post-infection (hpi). The percentage of NS5A-EGFP-positive cells was used to determine the HCV-Jc1EGFP infectivity. To determine the intracellular infectivity of HCVcc, HCV-infected cell pellets were resuspended in Dulbecco’s minimal essential medium containing 10% fetal bovine serum and then subjected to three cycles of freezing and thawing to induce cell lysis. The samples were subsequently centrifuged at 3,000 rpm for 5 min to remove cell debris; then, the supernatants were collected to determine the intracellular infectivity of HCVcc.

### RNA isolation, reverse transcription, and real-time PCR

RNA isolation was performed as previously described^26^. In brief, total intracellular RNA was extracted from the cells using TRIzol (Invitrogen) according to the manufacturer’s instructions. Extracellular HCV RNA was extracted from the supernatant using a QIAamp viral RNA mini kit (Qiagen). RNA was then reverse transcribed using a Moloney murine leukemia virus reverse transcriptase (Fermentas) in the presence of random hexamers (TaKaRa). The reaction mixture was incubated for 10 min at 25 °C and then for 1 h at 42 °C. The reaction was terminated by heating at 95 °C for 5 min. cDNA was used to perform SYBR-PCR using a SYBR Premix Ex Taq kit (TaKaRa). The housekeeping gene 18S rRNA was used as an internal control. The primer sequences used for real-time PCR are available upon request. Real-time PCR reactions were performed in triplicate using the CFX96 Touch™ real-time PCR detection system (BIO-RAD).

### Preparation of cell extracts and immunoblotting

Immunoblotting was performed as previously described^26^. Proteins were extracted from cells using radioimmunoprecipitation assay buffer supplemented with protease inhibitors (Cocktail, Sigma), and β-actin or GAPDH was used as an internal control. For immunoblotting, equal amounts of protein extracts were boiled in sodium dodecyl sulfate (SDS) loading buffer containing β-mercaptoethanol (140 mM) and then subjected to SDS-polyacrylamide gel electrophoresis. The electrophoresed samples were transferred onto PVDF (Millipore) or nitrocellulose membranes (Portran, Whatman) and incubated with the respective primary antibodies. Horseradish peroxidase-conjugated secondary antibodies were used to visualize the corresponding proteins in an enhanced chemiluminescence detection system (Millipore). Chemiluminescence light was detected using either a photographic film or a ChemiDoc™ MP system (Bio-Rad).

### Immunofluorescence assay (IFA) and confocal microscopy

Immunofluorescence assay and confocal microscopy experiments were performed as previously described^46^. Cells were seeded onto cover slides 24 h after transfection or 72 h after infection. After overnight culture, the cells seeded on the cover slides were directly fixed in 4% paraformaldehyde in PBS for 15 min at room temperature, followed by permeabilization with 0.5% Triton X-100 for 5 min. These cells were then stained with diluted antibodies. The FLAG epitope in the fusion proteins was detected using anti-FLAG monoclonal antibody M2 (Sigma, USA) followed by goat anti-mouse immunoglobulin conjugated to Alexa Fluor 555 dye (Beyotime). The HCV Core protein was detected using an anti-core mice monoclonal antibody followed by goat anti-mouse immunoglobulin conjugated with Alexa Fluor 555 dye. Endogenous CIDEB was detected using anti-CIDEB monoclonal antibody. APOE was detected using anti-APOE rabbit polyclonal antibody and Alexa Fluor 647-labeled goat anti-rabbit IgG (Beyotime) or Alexa Fluor 555-labeled donkey anti-rabbit IgG (H + L) (Beyotime). To counterstain nuclei, cells were incubated with 4′,6′-diamidino-2-phenylindole dihydrochloride (DAPI; Molecular Probes, USA). The cover slides were viewed and imaged using a confocal microscope (Carl Zeiss LSM 710 or Leica SP8 Confocal System).

### Immunoprecipitation (IP)

IP was performed as previously described^48^. In brief, transfected cells were washed with ice-cold phosphate-buffered saline (PBS), suspended in lysis buffer (50 mM Tris pH 7.4, 150 mM NaCl, 0.5% NP-40, 1.0 mM EDTA, and 0.5 mM PMSF) for 30 min at 4 °C, and passed 50 times through G23 needles. The lysates were centrifuged at 13,000 × *g* for 20 min at 4 °C. The bound antibody was added to the supernatant and incubated overnight at 4 °C. Approximately 80 μl of 50% protein A/G bead (Santa Cruz) suspension was added to the supernatant and subsequently incubated at 4 °C for 3 h. The beads were washed with lysis buffer once and with PBS five times. The beads were resuspended in 50 μl of PBS and then boiled with 12 μl of 5 × loading sample buffer for 10 min. The supernatant (25 μl per lane) was analyzed by SDS-polyacrylamide gel electrophoresis; the separated protein bands were transferred onto a nitrocellulose membrane (Portran, Whatman). The membrane was blocked for 1 h in PBS with 0.05% Tween-20 containing 5% milk and then incubated with antibody as needed. The bound antibodies were detected with horseradish peroxidase-conjugated rabbit anti-mouse IgG and enhanced chemiluminescence (Millipore).

### Yeast two-hybrid (Y2H)

The CIDEB–NS5A interaction was determined by Y2H analysis^48^. In brief, a panel of truncated mutants of CIDEB and NS5A was subcloned by PCR amplification. The mutants were inserted into pGBKT7 and pGADT7 by fusion with the DNA-binding and activation domains, respectively. Small-scale yeast mating was performed. AH109 yeast cells were pre-transformed with truncated NS5A in pGBKT7 and mated with Y187 yeast cells that were pre-transformed with truncated CIDEB in pGADT7. The mated yeast cells were then spread onto small SD agar plates, and positive clones were screened on SD/-Trp/-Leu/-His/-Ade and SD/-Trp/-Leu/-His plates with 2 mM 3-amino-124-triazole (3-AT, Sigma) at 30 °C for 5 to 8 d. AH109 yeast expressing pGBKT7-53 was mated with Y187 yeast expressing pGADT7-SV40T; the resulting product was used as a positive control. Empty pGBKT7 and pGADT7 were used as negative controls.

### Sucrose density gradient centrifugation

Sucrose density gradient centrifugation was performed as previously described^46^. The culture medium of HCV-infected cells treated with siRNAs was centrifuged (3,000 rpm, 20 min) to remove cellular debris and filtered through 0.45 μm filters. The supernatant was pelleted by centrifugation at 100,000 × *g* for 3 h at 4 °C. The pellet was resuspended in 300 μl of PBS buffer and applied to a 20–60% sucrose gradient (3.5 ml volume) in SW60 tubes (Beckman Coulter) and centrifuged at 100,000 × *g* (RCFav) for 16 h at 4 °C. We collected 340 μl fractions from the top of the gradient. The fractions were tested for protein levels using western blot , RNA levels using real-time PCR, and relative viral titer with limiting dilution assay.

### Statistical analysis

Data are presented as the mean ± standard deviations (SD) and were analyzed by *t*-test.

## Additional Information

**How to cite this article**: Cai, H. *et al*. Cell-death-inducing DFFA-like Effector B Contributes to the Assembly of Hepatitis C Virus (HCV) Particles and Interacts with HCV NS5A. *Sci. Rep*. **6**, 27778; doi: 10.1038/srep27778 (2016).

## Supplementary Material

Supplementary Information
