# Supplementary material for: Cell-death-inducing DFFA-like Effector B Contributes to the Assembly of Hepatitis C Virus (HCV) Particles and Interacts with HCV NS5A
Source: Sci Rep. 2016 Jun 10;6:27778. doi: 10.1038/srep27778 (PMC4901263; doi:10.1038/srep27778)
Supplement: Supplementary Information [file srep27778-s1.doc]

**Supplementary Material**

**Cell-death-inducing DFFA-like Effector B Contributes to the Assembly of Hepatitis C Virus (HCV) Particles and Interacts with HCV NS5A**

Hua Cai^†,1,2^, Wenxia Yao^†,1,2,*^, Leike Li^2,3^, Xinlei Li^1^, Longbo Hu^2,4^, Runming Mai^1^, Tao Peng^1,2,*^

^1^State Key Laboratory of Respiratory Disease, Guangzhou Hoffmann Institute of Immunology, College of Basic Sciences, Guangzhou Medical University, Guangzhou, China;

^2^Guangzhou Institutes of Biomedicine and Health, Chinese Academy of Sciences, Guangzhou, China;

^3^The Brown Foundation Institute of Molecular Medicine at the University of Texas Health Science Center at Houston, Houston, TX, USA;

^4^Division of Birth Cohort Study, Guangzhou Women and Children’s Medical Center, Guangzhou Medical University, Guangzhou, China;

^†^These authors contributed equally to this work.

^*^[Corresponding](http://dict.youdao.com/w/corresponding/) [author](http://dict.youdao.com/w/author/)s.

^*^Corresponding authors:

Wenxia Yao, Guangzhou Medical University, Guangzhou 511436, China. Tel: 86-20-37103810. E-mail: yaowenxia917@126.com

Tao Peng, Guangzhou Medical University, Guangzhou 511436, China. Tel: 86-20-37103810. E-mail: peng_tao@gibh.ac.cn

**Supplementary Figure S1**


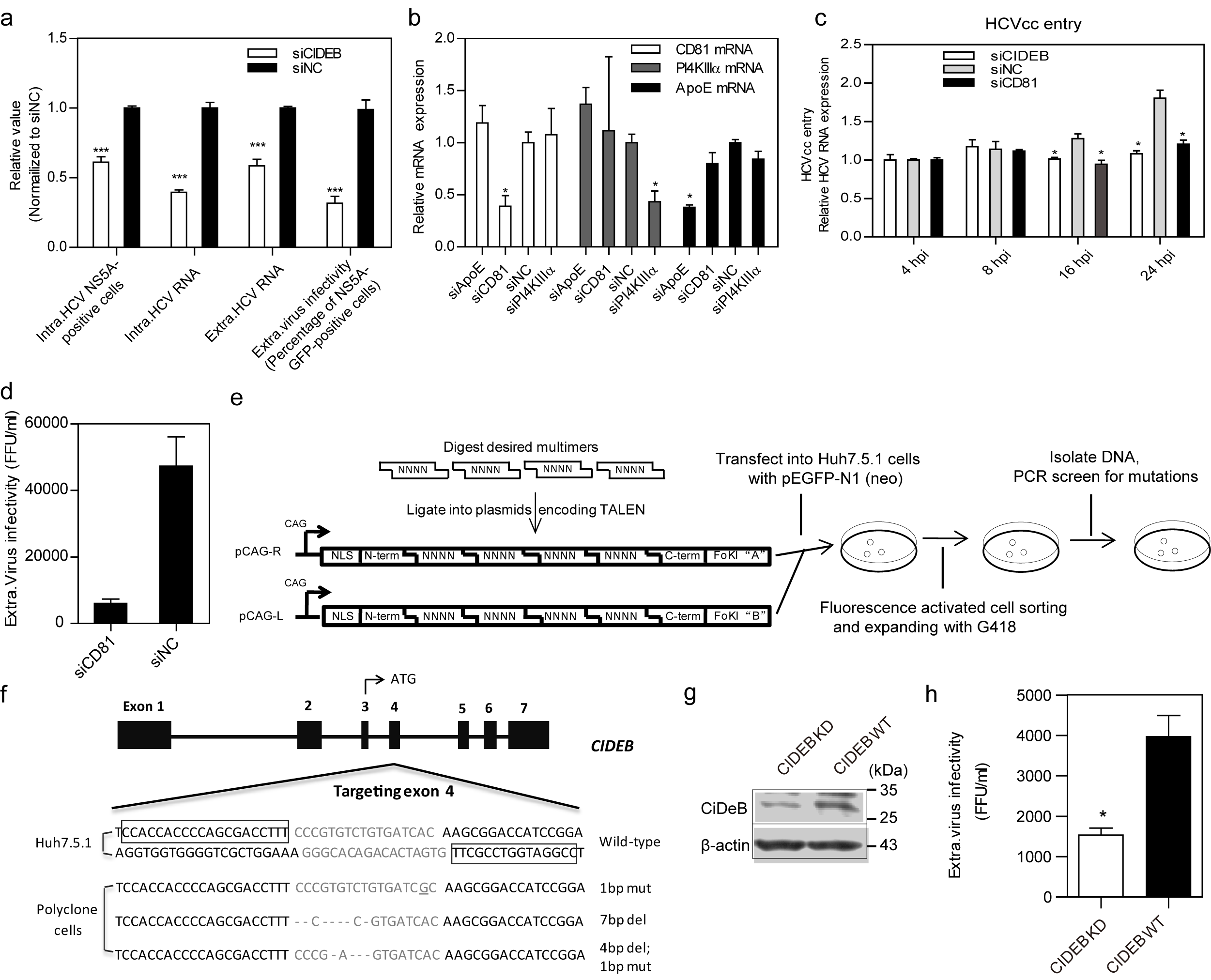


**Supplementary Figure S1.** **CIDEB is required for the assembly of HCV infectious particles.** (a) Effect of siCIDEB treatment on infectious HCV production. Huh7.5.1 cells were transfected with siCIDEB and then inoculated with HCV-Jc1EGFP (0.02 MOI) at 6 h post-transfection (hpt). Viral supernatants and cells were collected at 72 h post-infection (hpi). Viral spreading was quantified by FCM assay as a percentage of intracellular NS5A-EGFP-positive cells. Intracellular HCV RNA was quantified relative to 18S rRNA. Extracellular HCV RNA copies were calculated as genome equivalent (GE)/ml. Extracellular HCV infectivity was determined by flow cytometry (FCM). These values were normalized to the siNC control. (b) Cells were treated with siRNAs against the indicated genes. The knockdown efficiency of each siRNA was determined by quantifying the mRNA level of the target gene. (c) The effect of the knockdown of indicated genes on HCV entry was determined by time-course analysis. Huh7.5.1 cells were transfected with siRNA and then inoculated with HCV-Jc1EGFP at 48 hpt. The HCV RNA levels were further normalized to the value at 4 hpi after normalization to 18S rRNA. (d) The effect of siCD81 pre-treatment on HCV production was determined by comparing with naïve Huh7.5.1 cells infected with HCV-Jc1EGFP. (e-h) A TALEN pair was designed to determine the effect of CIDEB knockdown on HCV infection. (e) Schematic of the system for efficient and rapid genome editing using TALEN. (f) Generation of CIDEB knockdown Huh7.5.1 polyclone cells with TALENs targeting exon 4 of CIDEB. The boxes indicate TALEN binding sites. Mutations and deletions in the alleles of the polyclonal cells are indicated. (g) CIDEB protein levels in wild-type (WT) and CIDEB-knockdown (KD) Huh7.5.1 cells were detected by Western blot. (h) Persistently HCV-infected Huh7.5.1 cells were transfected with the CIDEB TALEN pair or Control TALEN pair, and the HCV titer in the supernatants was then evaluated at 4 dpt. (a, b, c and h) The results are presented as the mean ± SD (*n ≥* 3 independent experiments; *P < 0.05, **P < 0.01,***P < 0.001).

**Supplementary Figure S2**


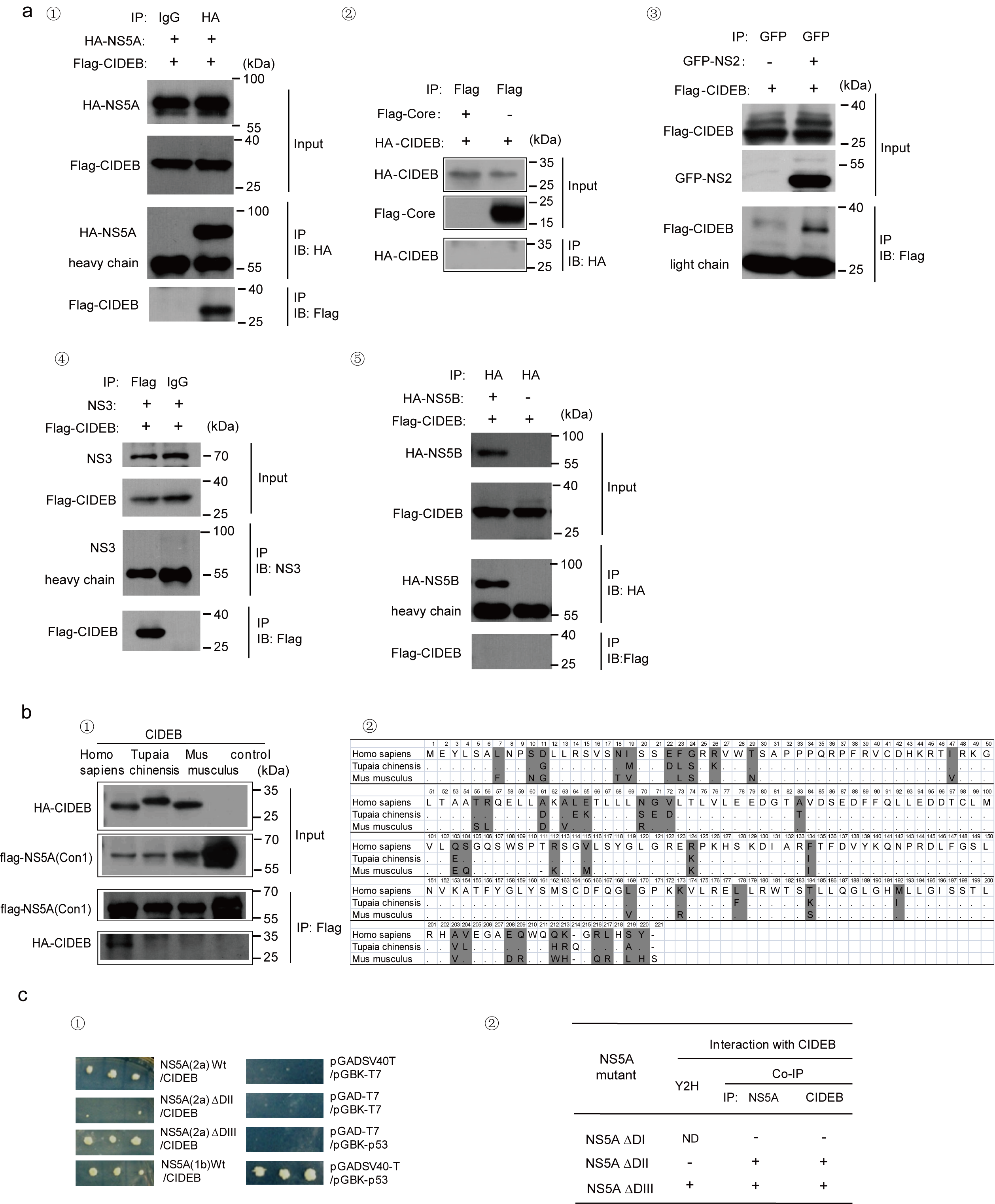


**Supplementary Figure S2. CIDEB interacts with NS5A.** (a) Co-IP assays to determine the interactions of exogenous CIDEB with HCV proteins (①NS5A, ②Core, ③NS2, ④NS3, and ⑤NS5B) in HEK293T cells. The cell lysates were subjected to IP with an anti-HA antibody, anti-Flag antibody, anti-GFP antibody or IgG control and then immunoblotted with an anti-Flag, anti-HA antibody or anti-NS3 antibody. (b) Left: Co-IP assay to determine the interaction of exogenous CIDEBs from different species with Flag-NS5A from Con1 (genotype 1b) in HEK293T cells. Right: Manual sequence alignment of CIDEB from different species including *Homo sapiens*, *Tupaia chinensis*, and *Mus musculus*. (c) Left: Screening results of the interaction of truncated NS5A with CIDEB by yeast two-hybrid (Y2H) assay. Right: Summary of the specific domain of NS5A responsible for the CIDEB-NS5A interaction in Y2H and co-IP assays.

**Supplementary Figure S3**

**
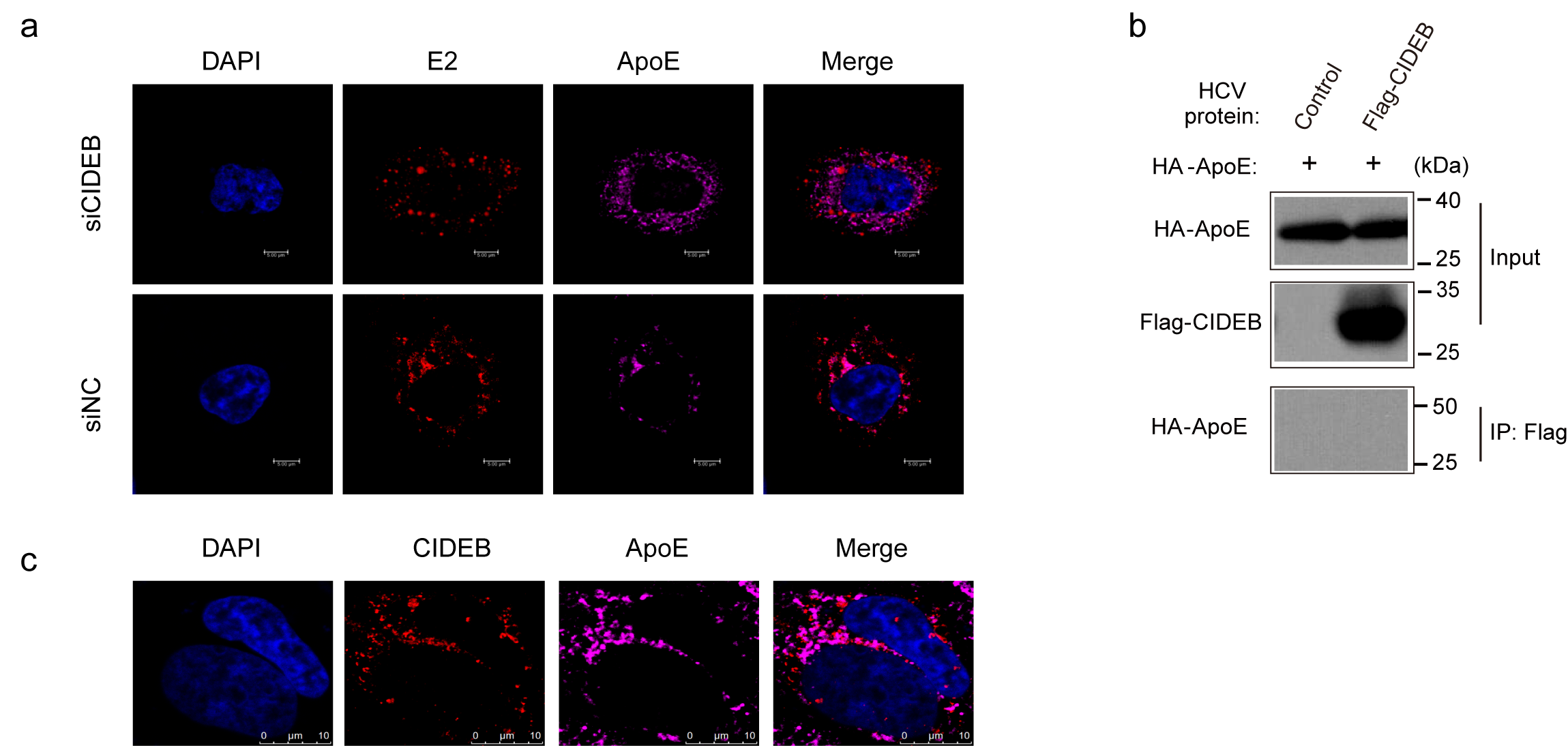
**

**Supplementary Figure S3. The effect of CIDEB silencing on the co-localization of E2 with APOE.** (a) The effect of CIDEB silencing on the co-localization of E2 with ApoE in HCV persistently infected Huh7.5.1 cells. (b) Co-IP assays to determine the interaction of exogenous Flag-CIDEB with HA-tagged ApoE in HEK293T cells. Cell lysates were subjected to IP with an anti-Flag antibody and then immunoblotted with an anti-HA antibody. (c) The co-localization of CIDEB with ApoE was analyzed by confocal microscopy.
